# Comparing the concentration levels of allergens and endotoxins in employees’ homes and offices

**DOI:** 10.1007/s00420-021-01794-9

**Published:** 2021-11-05

**Authors:** Ingrid Sander, Anne Lotz, Verena Liebers, Eva Zahradnik, Ulrich Sauke-Gensow, Jens Petersen, Monika Raulf

**Affiliations:** 1 Institute for Prevention and Occupational Medicine, German Social Accident Insurance, Institute of the Ruhr University Bochum (IPA), Bürkle-de-la-Camp-Platz 1, 44789 Bochum, Germany; 2 Verwaltungsberufsgenossenschaft (VBG), German Social Accident Insurance, Hamburg, Germany

**Keywords:** Cat allergen Fel d 1, Dog allergen Can f 1, Domestic mite allergens, Electrostatic dust collectors, Endotoxin

## Abstract

**Objective:**

The aim of the study was to find out whether allergen and endotoxin concentrations in offices differ from those measured at the homes of employees, and identify the parameters that influence exposure.

**Methods:**

Electrostatic dust collectors (EDCs) were placed in five office buildings (68 rooms, 436 EDCs), as well as the homes of the office workers (145 rooms, 405 EDCs) for 14 days, four times a year. In addition, surface samples were collected from the offices four times a year by vacuuming the carpeted floors. Domestic mite (DM), and the major cat and dog allergens (Fel d 1 and Can f 1) were quantified in all samples using fluorescence enzyme immunoassays. Endotoxin was measured in the EDC samples, using the Limulus amoebocyte lysate assay. The allergen and endotoxin concentrations were log transformed and analysed with multilevel models.

**Results:**

Endotoxin concentrations were significantly higher in personal homes compared to levels measured in the offices, and depended on the number of persons living in each household, as well as the presence of a dog. DM allergens were significantly higher in households than in offices, and were significantly higher in bedrooms compared to living rooms. Offices occupied by cat owners had significantly higher Fel d 1 concentrations than offices or homes without. Additionally, Can f 1 concentrations were significantly higher in offices occupied by dog owners compared to those without.

**Conclusions:**

Pet owners appear to transfer cat and dog allergens to their offices. Therefore, in case of allergy complaints at the office, employers and physicians might consider possible contamination by cat and dog allergens.

**Supplementary Information:**

The online version contains supplementary material available at 10.1007/s00420-021-01794-9.

## Introduction

Allergies affect an increasing number of people, especially in industrialised countries, and allergy development is a growing concern at various workplaces. Exposure to allergens and microbial agents is known to influence respiratory health (Sigsgaard et al. 2020; Liebers et al. 2020). The risk of developing occupational asthma is increased when working with laboratory animals or in bakeries. However, besides specific workplace allergens (Baur and Bakehe 2014), there are indications that environmental allergens are also elevated at the workplace, causing sensitization and complaints. Recent studies in day-care centres and schools, where electrostatic dust collectors (EDCs) were used for exposure detection, found high concentrations of cat and dog allergens, as well as domestic mite allergens (Krop et al. 2014; Sander et al. 2018). Furthermore, endotoxin concentrations at schools were higher than those measured in the home environment, and were associated with airway inflammation and an increased risk of non-atopic respiratory diseases (Jacobs et al. 2013; Lai et al. 2015).

Currently, there is growing concern about indoor air quality and its impact on the health, comfort and work performance of office workers, who make up the majority of employees in many industrialised countries (Carrer and Wolkoff 2018). But, there are only a few studies that have measured allergen and endotoxin concentrations in offices. Brunetto et al.(2009) used monoclonal antibody-based immunoassays to investigate single house dust mite and cat allergens on floor and furniture surfaces of offices, schools and homes. They found house dust mite allergens in only a few samples from offices and schools (< 10%), and in the majority of the samples collected from homes. Conversely, cat allergen was measured at a similar frequency (64–69%) in all environments. Studies from Malaysia revealed that Der f 1 allergen level in office dust was a risk factor for daytime breathlessness among office workers (Lim et al. 2015), and endotoxin was a risk factor for wheezing and rhinoconjunctivitis (Lim et al. 2019). Furthermore, cat and dust mite allergens were frequently detected in surface samples collected from US office buildings (Macher et al. 2005), but house dust mite allergen levels were low in most cases, and only for a subgroup of office workers higher than 1 µg/g floor dust and related to work-related respiratory tract symptoms (Menzies et al. 1998). These studies all used surface dust samples for exposure assessment, which might be a poor correlate to concentrations in airborne samples. This limitation can be reduced by collecting samples using EDCs, in addition to using particularly sensitive allergen quantification methods. As these methods have not been used in offices so far, we conducted a study on endotoxin and allergen concentration on EDCs in offices in Germany. For comparison, samples were simultaneously taken from the households of employees. Sampling started in the spring and was repeated in summer, autumn and winter in 67 office rooms and 145 rooms of the employees’ homes. The aim of the study was to find out (1) whether the allergen and endotoxin in offices differ from those in the homes of employees, (2) identify parameters that influence the allergen and endotoxin concentrations in offices, and (3) the parameters influencing allergen and endotoxin concentrations in homes.

## Methods

### Sampling and and extraction methods

Sample collection was carried out at five companies with offices in Hamburg and Berlin, starting in Spring 2015 until Winter 2016. Permission for sample collection at the workplace was granted by the employers, the staff council and the employees involved. An ethics committee was not involved as no clinical or personal data was collected. Besides an open-plan office with 450 workstations, there were 29 individual offices, 28 with 2–4 and 10 with 5–28 workstations. In these 68 offices, surface samples were taken four times a year by vacuuming 0.4 m^2^ areas on the carpeted floors using rectangular templates. In large rooms (≥ 150 m^2^ or more than 6 employees), more than one sample was taken per room at each timepoint (altogether 437 samples). In addition, EDC were laid out for 14 days with the same number and frequency in the 68 offices (436 samples), and in parallel in the 145 households of volunteers from the companies (405 samples). The consent of the volunteers resulted from the return of the questionnaires and EDCs. The questionaires, sample collection by vacuuming and the EDC sampling method had been validated during our previous studies (Sander et al. 2016a, 2016b, 2018). Of the volunteers, 30 (20.4%) had cats and 14 (9.5%) had dogs at home.

**Fig. 1:**
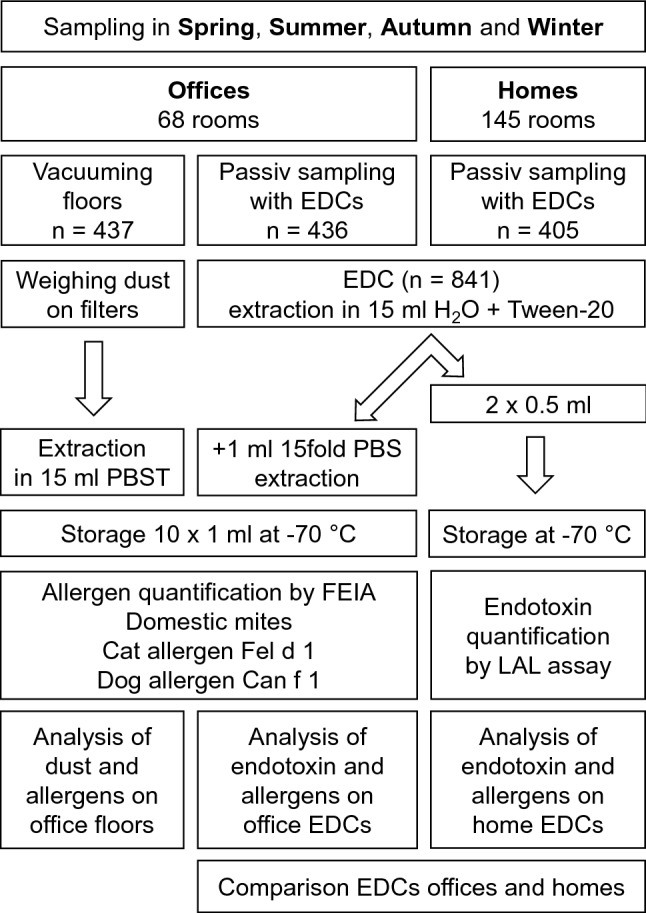
Overview of the sample collection, processing and analysis

The sampling procedure, weighing of dust, and extraction method were the same as previously described for our study in day-care centres (Sander et al. 2016b, 2018). However, the EDC extraction was adapted for the additional measurement of endotoxin. Endotoxin trapped in EDC cloths was extracted in 15 ml pyrogen-free water with 0.05% Tween 20 and aliquots of 2 × 0.5 ml of the supernatant after centrifugation at 3000 ×*g* were removed. Then 1 ml of 15 fold phosphate buffered saline (PBS) was added, allergens were extracted and the supernatant was stored in 10 × 1 ml aliquots. Figure 1 shows the sample collection and processing procedure schematically.

Questionnaires were used to record room characteristics, as well as to obtain data on use, cleaning and pet ownership. The office questionnares were completed by the field worker on the days of sampling. Similar questionnaires had been used in our previous study in day-care centres (Sander et al. 2016b, 2018). Relative air humidity was measured at the time of each visit to the offices and reached from 19.5.to 73%.

### Allergen quantification with fluorescence enzyme immunoassays (FEIAs)

Domestic mite (DM), cat (Fel d 1), and dog (Can f 1) allergens were quantified by FEIAs as described previously (Sander et al. 2016b). The DM FEIA is based on polyclonal antibodies to *Dermatophagoides farinae* (Sander et al. 2012). Monoclonal antibodies and multiallergen standards (Filep et al. 2012) were purchased from Indoor Biotechnologies Ltd. (Charlottesville, VA). The kit, EL-FD1, was employed to measure Fel d 1. For detection of Can f 1, the biotinylated monoclonal antibody 6E9 was used for detection and the monoclonal antibody 10D4 was utilized as the capture antibody. To compare the data, the same fixed detection limits (LOD) were used as in the study conducted in day-care facilities (DM 0.05 ng/ml, Fel d 1 and Can f 1 0.01 ng/ml) (Sander et al. 2018, 2016b), but a different strategy was applied for the values below these limits. The values were calculated using the four-parameter fit of the standard curve as far as possible.

In the vacuumed floor samples, dust and allergen concentrations were above the LOD with the exception of three samples in which Can f 1 level were below the LOD. In the 841 EDC samples, 561 (66.7%) had DM allergen concentrations above the LOD and 699 (83.1%) were in the range of the standard curve, 408 (48.5%) had Fel d 1 concentrations above the LOD and 738 (87.8%) were in the range of the standard curve, 237 (28.2%) had Can f 1 concentrations above the LOD and 441 (52.4%) were in the range of the standard curve.

### Endotoxin quantification in EDC samples

For the detection of endotoxin (activity expressed as endotoxin units = EU), the chromogen-kinetic LAL test Endosafe Endochrome-K (Charles River, Sulzfeld, Germany) was used. One lot of control standard endotoxin (CSE; E. coli O55:B5) was used for all tests (Lot nr. EX64062, 8 EU/ng). Lyophilised CSE was dissolved in *Aqua injectabilia* with 0.05% Tween-20, and reconstituted to a concentration of 50 EU/ml. EDC sample extracts were thawed and diluted with pyrogen-free water with 0.05% Tween 1:10 before measurement. 100 µl of the sample were incubated for 10 min at 20 °C in a 96-well microplate (Falcon 3072, Becton Dickinson, Heidelberg, Germany). The LAL reagent was rapidly added to the samples and kinetics were recorded at 405 nm and 37 °C using a temperature controlled microplate reader (SpectraMax 340PC and software Softmax Pro 5.4.6, Molecular Devices, Sunnyvale, USA). For each measurement, a fresh standard curve in the range from 0.005 to 50 EU/ml was prepared.

As controls, all samples were spiked with CSE (5 EU/ml final activity). The recovery of spiked samples was in the required range of 50–200% (median 108%). All EDC samples were measurable.

### Statistical analysis

The allergen and endotoxin concentrations were log transformed and modelled using multilevel-level models with sample as level-one unit and room as level-two unit to take dependencies of the samples from the same room into account. We did not consider the company building as level-three unit, because the models considered the explanatory variable “ventilation, renovation and cleaning”, which occurred only in certain combinations and was linked to the building. If some of the concentrations were below the standard curve, the multilevel-level models were estimated following the method of Vaida and Liu (2009). To model the allergen and endotoxin concentrations at the offices and at homes, a forward selection method based on Akaike information criterion (AIC) was applied to select a set of explanatory variables without considering interaction terms. All independent variables including the information of being between- or within-room variables and sample numbers in office models are in S1 Table; and similarly the variables and sample numbers in employees’ homes are in S2 Table. The selection of additional variables stopped when no further reduction in AIC was achieved.

To estimate the fraction of total variation that is accounted for by between-room variation conditional on the fixed effects, conditional intraclass correlation coefficients (ICC) of the full model were calculated. The ICC approaches one, when the between-room variation is very large relative to the within-room variation indicating that samples taken in the same room are similar. In contrast, the ICC approaches zero when there is no multilevel-effect and the grouping of samples by room conveys no additional information.

Missing values in the independent variables were supplemented either by information known for another sample (e.g. sample from the same room at another season) or by the mode value. *P* values < 0.05 were considered statistically significant, though it should beared in mind that no correction for multiple comparisons was applied due to the exploratory nature of the analysis. The statistical analysis was performed with SAS, version 9.4 (SAS Institute, Inc. Cary, NC). The multilevel models with a censored dependent variable were calculated in R, version 4.0.0 (R Core Team 2015). The graphs were made with GraphPad Prism, version 8.4.3 (GraphPad Software, Inc., La Jolla, CA). In figures, values below the range of the standard curve were set to the minimum value of the data set.

## Results

### Allergen and endotoxin levels from EDCs in offices and dwellings

**Fig. 2:**
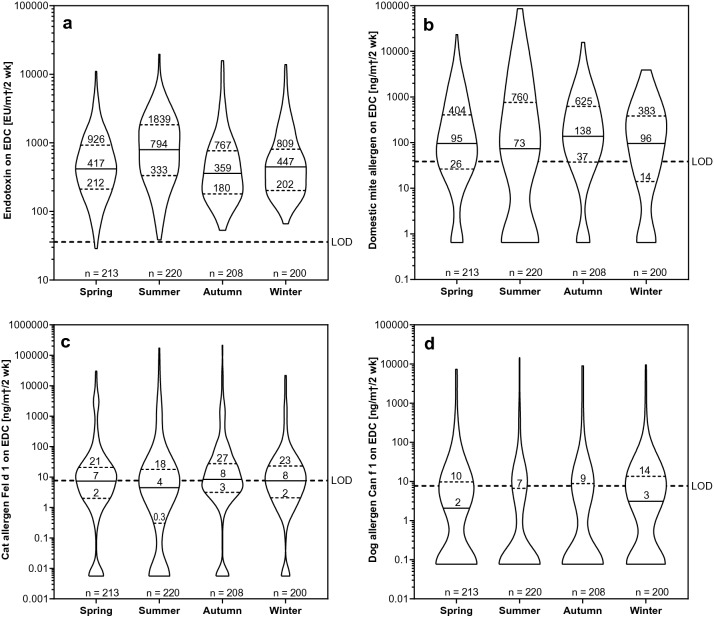
Influence of the season on allergen and endotoxin concentrations. Violin plots of **a** endotoxin, **b** domestic mite allergen DM, **c** cat allergen Fel d 1 and **d** dog allergen Can f 1 concentrations in electrostatic dust collector (EDC) samples from offices and dwellings combined, sorted according to season, including median (solid line) and interquartile ranges (dotted lines). Fixed detection limits (LOD) are indicated by dashed lines. In addition, the number of samples is given for each season

**Fig. 3:**
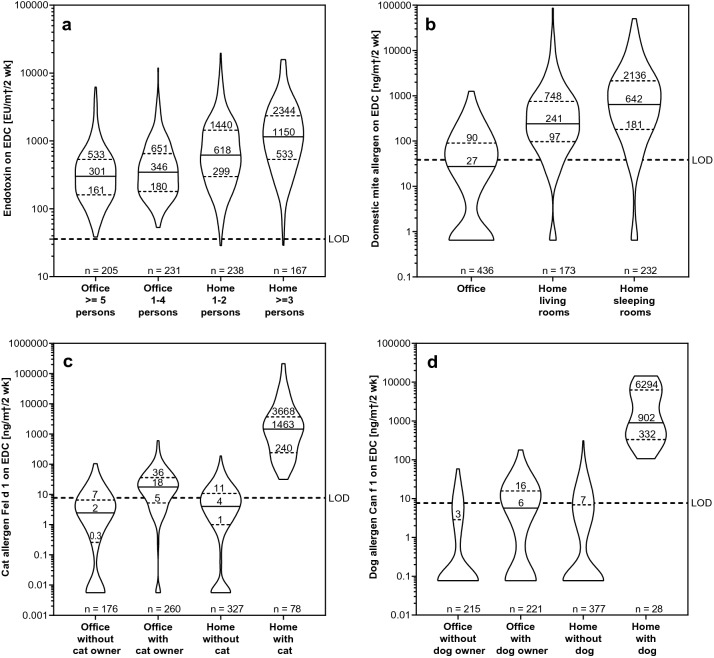
Important parameters influencing concentrations in offices and employees’ homes. Violin plots of EDC samples and **a** endotoxin concentrations sorted by number of persons per room in offices and home, **b** DM concentrations in offices and living and sleeping rooms in homes, **c** Fel d 1 and **d** Can f 1 concentrations according to pet ownership including median (solid line) and interquartile ranges (dotted lines)

In the statistical models for all EDC samples from offices and households, only the most important measurement parameters were taken into account. These included the season for endotoxin and all allergens (Fig. 2), the number of persons in the room for endotoxins, the room use in households (living or sleeping) for DM, and pet ownership for Fel d 1 and Can f 1 (Fig. 3). The measurement results are shown graphically using violin plots that show in addition to median and interquartile ranges (IQR) the different distributions of the concentrations. The statistical models are shown in the supplementary material (S3 Tables) (Fig. 3).

Endotoxin concentrations were significantly higher in summer compared to the other seasons (*p* < 0.0001); whereas, DM and Fel d 1 concentrations were highest in autumn, and Can f 1 concentrations significantly higher in winter compared to the other seasons (*p* < 0.0001–0.044). Endotoxin concentrations were found to be significantly higher in dwellings than in the offices (*p* < 0.0001–0.0485), and were dependent on the number of persons in the households. Rooms with three or more persons had significantly higher concentrations than rooms with one or two persons (*p* < 0.0001). DM allergens were significantly higher in households than in offices (*p* < 0.0001), and were significantly higher in bedrooms compared to living rooms (*p* = 0.0085). Cat and dog allergen concentrations were significantly higher in the homes where these pets were present compared to homes without, as well as the offices (*p* < 0.0001). Furthermore, offices with cat owners had a significantly higher Fel d 1 concentration than offices and homes without cat owners (*p* < 0.0001 and *p* = 0.0004, respectively). Finally, offices occupied by dog owners had significantly higher Can f 1 allergen levels compared to offices without dog owners (*p* = 0.0007); however, the difference to dwellings without dogs was not significant (*p* = 0.156).

### Influences on dust, endotoxin and allergen levels in offices

In offices, samples were acquired either by vacuuming the floors or by passive sampling with the EDC. From the floor samples, both allergens and the amount of dust were quantified; whereas, in the EDC samples, allergens and the endotoxin levels were measured. Using forward selection, the parameters with the greatest influence on the concentrations were selected and considered in the final models (Tables 1, 2, 3, 4). For the dust concentration, only the season, number of people working in the room, and cleaning, ventilation and renovation had a significant influence (Table 1). The room parameters were only present in certain combinations in the five companies, making it difficult to determine the individual influence of renovation, cleaning or ventilation system. However, it appears that cleaning every day, or every 1 or 2 days reduces the dust concentration; whereas, a ventilation system had the opposite effect. With respect to DM, there was a similar trend for floor cleaning, but the ventilation system did not have an adverse effect. Regarding animal allergens, the presence of a pet owner in the office had the strongest effect on the allergen concentration (Table 2). In addition, for Fel d 1 there was also a significantly increasing effect in case of customer contact. A pet owner in the office led to an even more pronounced effect on the Fel d 1 and Can f 1 concentrations in the models for the EDC samples (Table 4). The concentration of DM allergens increased primarily in autumn, and in rooms with air conditioning or ventilation systems (Table 3). In contrast, endotoxin was highest in winter and spring, and in rooms with window ventilation. Higher relative humidity increased the endotoxin levels. The conditional ICC of endotoxin and DM was only 12% and 24%, respectively (Table 3). Thus, a specific room did not seem to be a robust or dominant determinant of endotoxin and DM concentrations on EDCs. Conversely, a specific room was a main determinant regarding the Fel d 1 (ICC 62%) and Can f 1 (ICC 66%) concentrations on EDCs (Table 4), and had a moderate influence on dust and allergen concentrations in the floor samples (ICC 35–44%, Table 1, 2).

**Table 1:** Multilevel models of dust and DM levels on carpeted floors in offices

Fixed effects		Number (*n*)	Dust				Domestic mite			
Variable	Category		Median (IQR) [mg/m^2^]	Estimate	95% CI	p value	Median (IQR) [ng/m^2^]	Estimate	95% CI	*p* value
Intercept				170	(135–214)			273	(115–649)	
Season	Spring	107^a^	87 (29–185)	1			80 (34–195)	1		
	Summer	110	73 (40–166)	1.11	(0.98–1.26)	0.1	68 (35–163)	0.9	(0.76–1.08)	0.26
	Autumn	110	76 (37–162)	1.08	(0.95–1.22)	0.26	123 (76–264)	1.74	(1.46–2.07)	** < 0.0001**
	Winter	110	89 (41–196)	1.25	(1.10–1.42)	**0.0005**	100 (57–305)	1.52	(1.27–1.80)	** < 0.0001**
Number of employees in room	1	124^a^	121 (67–218)	1			147 (68–342)	1		
	2–4	108	164 (88–233)	1.11	(0.87–1.42)	0.39	187 (95–381)	0.94	(0.65–1.36)	0.74
	5–24	72	126 (74–177)	0.65	(0.43–0.97)	**0.033**	95 (47–137)	0.47	(0.26–0.87)	**0.016**
	450	133	30 (22–42)	0.48	(0.15–1.49)	0.2	57 (33–83)	0.32	(0.05–2.20)	0.25
Customer contact	No	181					63 (33–104)	1		
	Yes	256					144 (73–322)	0.97	(0.43–2.18)	0.94
Ventilation, renovation and cleaning	Windows to open. cleaning weekly. no floor renovation	116	193 (134–249)	1			295 (159–480)	1		
	Windows to open. cleaning dayly. no flour renovation	28	164 (93–222)	0.80	(0.55–1.16)	0.24	239 (102–399)	0.59	(0.29–1.21)	0.15
	Windows to open. cleaning 1–2 days ago. floor renovation	72^a^	70 (45–94)	0.37	(0.28–0.48)	** < 0.0001**	64 (36–123)	0.22	(0.15–0.33)	** < 0.0001**
	Air conditioning. cleaning daily. no floor renovation	20	21 (11–41)	0.13	(0.07–0.24)	** < 0.0001**	34 (28–69)	0.19	(0.06–0.65)	**0.0082**
	Air conditioning. cleaning daily. floor renovation	137	31 (22–43)	0.32	(0.13–0.78)	**0.012**	60 (34–87)	0.49	(0.13–1.88)	0.29
	Ventilation system. cleaning 1–2 days ago. no floor renovation	64	158 (101–250)	1.13	(0.79–1.64)	0.5	116 (67–173)	0.57	(0.32–1.00)	0.051

Random effects	Dust	Domestic mite
Level-one variance (Sample)	0.23	0.42
Level-two variance (Room)	0.14	0.34
Conditional ICC	0.38	0.44

^a^In case of dust one sample less. Influences with *p* values < 0.05 are printed in bold font.* IQR* Interquartile range. Estimate: This is the transformed coefficient estimate exp (β) of the multilevel model. Conditional *ICC* The conditional intraclass correlation coefficient is controlled for covariatess

**Table 2:** Multilevel models of cat and dog allergen levels on carpeted floors in offices

Fixed effects		Number (*n*)	Fel d 1				Can f 1			
Variable	Category		Median (IQR) [ng/m^2^]	Estimate	95% CI	*p* value	Median (IQR) [ng/m^2^]	Estimate	95% CI	*p* value
Intercept				5.47	(2.11–14.20)			2.28	(0.78–6.71)	
Season	Spring	107	17.1 (7.1–30.2)	1			5.0 (2.5–10.2)	1		
	Summer	110	16.8 (8.6–29.9)	1	(0.80–1.25)	0.99	3.4 (1.7–6.2)	0.65	(0.53–0.81)	** < 0.0001**
	Autumn	110	17.6 (10.8–31.9)	1.05	(0.84–1.31)	0.65	5.5 (3.3–9.0)	1.09	(0.88–1.35)	0.41
	Winter	110	18.2 (9.4–37.2)	1.03	(0.83–1.28)	0.78	7.7 (3.3–13.8)	1.24	(1.00–1.53)	0.049
Number of employees in room	1	124	14.9 (5.4–28.2)	1			3.6 (1.7–7.7)	1		
	2–4	108	17.0 (8.4–36.7)	0.95	(0.63–1.43)	0.81	4.9 (2.0–11.7)	1.09	(0.69–1.71)	0.72
	5–24	72	17.3 (9.8–33.2)	0.44	(0.21–0.92)	**0.028**	6.5 (3.6–14.1)	1.11	(0.49–2.52)	0.81
	450	133	19.7 (12.5–35.0)	1.49	(0.18–12.37)	0.71	5.9 (3.3–10.5)	0.53	(0.04–6.48)	0.62
≥ 1 employee with cat	No	176	12.5 (5.4–26.4)	1			4.8 (2.5–9.9)	1		
	Yes	261	19.7 (12.1–38.6)	3.12	(1.92–5.05)	** < 0.0001**	5.3 (2.4–10.2)	0.72	(0.43–1.23)	0.23
≥ 1 employee with dog	No	216					3.5 (1.8–8.0)	1		
	Yes	221					6.2 (3.6–12.5)	3.42	(1.87–6.26)	** < 0.0001**
Customer contact	No	181	18.1 (10.9–30.6)	1			5.4 (2.9–10.0)	1		
	Yes	256	16.7 (7.6–35.4)	3.19	(1.32–7.75)	**0.01**	5.0 (2.3–9.9	2.03	(0.76–5.43)	0.16
Height of room	< 3 m	236					4.7 (2.3–8.5)	1		
	> 3 m	201					5.6 (2.9–11.4)	1.95	(1.14–3.32)	**0.015**
Ventilation, renovation and cleaning	Windows to open, cleaning weekly, no floor renovation	116	21.1 (9.8–44.3)	1			5.7 (2.9–11.9)	1		
	Windows to open, cleaning daily, no flour renovation	28	10.7 (5.0–23.3)	1.15	(0.52–2.55)	0.72	3.2 (1.3–6.5)	0.92	(0.38–2.24)	0.86
	Windows to open, cleaning 1–2 days ago, floor renovation	72	7.7 (3.8–19.2)	0.34	(0.22–0.54)	** < 0.0001**	2.4 (1.4–5.7)	0.55	(0.33–0.91)	**0.019**
	Air conditioning, cleaning daily, no floor renovation	20	15.1 (9.1–12.5)	1.42	(0.37–5.42)	0.61	3.4 (1.9- 7.0)	0.27	(0.05–1.35)	0.11
	Air conditioning, cleaning daily, floor renovation	137	19.7 (12.5–34.4)	0.85	(0.19–3.77)	0.83	5.6 (3.3–10.4)	1.01	(0.20–5.18)	0.99
	Ventilation system, cleaning 1–2 days ago, no floor renovation	64	17.6 (10.4–30.3)	0.87	(0.46–1.63)	0.65	7 (3.8–16.1)	1.1	(0.54–2.27)	0.79

Random effects	Fel d 1	Can f 1
Level-one variance (Sample)	0.67	0.63
Level-two variance (Room)	0.37	0.49
Conditional ICC	0.35	0.44

Influences with *p* values < 0.05 are printed in bold font. *IQR* Interquartile range. Estimate: This is the transformed coefficient estimate exp (β) of the multilevel model. Conditional *ICC* The conditional intraclass correlation coefficient is controlled for covariates

**Table 3:** Multilevel linear models of endotoxin and DM levels on EDCs in offices

Fixed effects	Category	Number (*n*)	Endotoxin				Domestic mite			
Variable			Median (IQR) [EU/m^2^/2 week]	Estimate	95% CI	*p* value	Median (IQR) [ng/m^2^/2 week]	Estimate	95% CI	*p* value
Intercept				192	(129–285)			40.8	(18–90)	
Season	Spring	106	374 (182–689)	1			33.5 (13.1–75.4)	1		
	Summer	110	395 (190–769)	0.74	(0.58–0.96)	**0.024**	< 0.6 (< 0.6–42.3)	0.07	(0.03–0.14)	** < 0.0001**
	Autumn	110	245 (148–466)	0.59	(0.47–0.74)	** < 0.0001**	63.8 (16.1–161.5)	1.29	(0.64–2.58)	0.47
	Winter	110	267 (170–502)	1.03	(0.83–1.29)	0.77	22.3 (< 0.6–88.4)	0.44	(0.22–0.89)	**0.02**
Number of employees in room	1	123	360 (169–682)	1						
	2–4	108	337 (200–626)	1.1	(0.84–1.44)	0.5				
	5–24	72	524 (294–822)	1.8	(1.17–2.78)	**0.0081**				
	450	133	220 (141–373)	1.83	(0.59–5.68)	0.29				
Air humidity	(per 1% increase)			1.04	(1.02–1.05)	** < 0.0001**				
≥ 1 employee with dog	No	215					26.9 (< 0.6–99.0)	1		
	Yes	221					28.7 (< 0.6–86.9)	0.22	(0.06–0.76)	**0.017**
Ventilation, renovation and cleaning	Windows to open, cleaning weekly, no floor renovation	116	397 (183–746)	1			39.2 (< 0.7–113.8)	1		
	Windows to open, cleaning daily, no flour renovation	27	456 (200–713)	0.99	(0.65–1.52)	0.98	26.6 (< 0.6–99.0)	0.46	(0.10–2.08)	0.31
	Windows to open, cleaning 1–2 days ago, floor renovation	72	353 (205–686)	1.01	(0.75–1.37)	0.93	4.2 (< 0.6–27.9)	0.11	(0.04–0.33)	**0.0001**
	Air conditioning, cleaning daily, no floor renovation	20	287 (123–714)	0.57	(0.31–1.04)	0.067	29.2 (< 0.6–77.8)	2.11	(0.19–23.4)	0.54
	Air conditioning, cleaning daily, floor renovation	137	214 (141–372)	0.33	(0.12–0.88)	**0.027**	32.3 (< 0.6–93.8)	2.51	(0.28–22.6)	0.41
	Ventilation system, cleaning 1–2 days ago, no floor renovation	64	422 (270–727)	0.95	(0.63–1.43)	0.81	38.8 (4.2–91.5)	1.5	(0.38–5.93)	0.56

Random effects	Endotoxin	Domestic mite
Level-one variance (Sample)	0.62	6.56
Level-two variance (Room)	0.09	2.08
Conditional ICC	0.12	0.24

Influences with *p* values < 0.05 are printed in bold font. *IQR* Interquartile range. Estimate: This is the transformed coefficient estimate exp (β) of the multilevel model. Conditional *ICC* The conditional intraclass correlation coefficient is controlled for covariates

**Table 4:** Multilevel linear models of cat and dog allergen levels on EDCs in offices

Fixed effects	Category	Number (*n*)	Fel d 1				Can f 1			
Variable			Median (IQR) [ng/m^2^/2 week]	Estimate	95% CI	*p* value	Median (IQR) [ng/m^2^/2 week]	Estimate	95% CI	*p* value
Intercept				4.15	(0.49–35)			0.04	(0.02–0.12)	
Season	Spring	106	7.7 (2.3–20.0)	1			1.8 (< 0.1–10.8)	1		
	Summer	110	3.7 (0.2–16.1)	0.27	(0.15–0.48)	** < 0.0001**	< 0.1 (< 0.1–1.8)	0.07	(0.02–0.18)	** < 0.0001**
	Autumn	110	11.5 (4.1–26.1)	2.11	(1.18–3.77)	**0.012**	< 0.1 (< 0.1–6.8)	0.32	(0.12–0.85)	**0.0227**
	Winter	110	9.3 (2.5–25.4)	1.45	(0.81–2.60)	0.21	5.5 (< 0.1–16.1)	2.68	(1.04–6.86)	**0.0404**
Occupancy (quotient)				0.98	(0.95–1.00)	0.059				
≥ 1 employee with cat	No	176	2.5 (0.3–6.5)	1						
	Yes	260	17.7 (5.3–35.8)	8.82	(3.26–23.8)	** < 0.0001**				
≥ 1 employee with dog	No	215					< 0.1 (< 0.1–2.9)	1		
	Yes	221					5.7 (< 0.1–15.4)	17.20	(3.52–84.08)	**0.0006**
Height of room	< 3 m	235	4.2 (1.0–13.8)	1			< 0.1 (< 0.1–2.9)	1.00		
	> 3 m	201	14.6 (4.6–35.4)	3.12	(0.99–9.79)	0.052	3.9 (< 0.1–13.8)	3.90	(1.19–29.16)	**0.0295**

Random effects	Fel d 1	Can f 1
Level-one variance (Sample)	4.67	10.70
Level-two variance (Room)	7.66	20.68
Conditional ICC	0.62	0.66

Influences with *p* values < 0.05 are printed in bold font. *IQR* Interquartile range. Estimate: This is the transformed coefficient estimate exp (β) of the multilevel model. Conditional *ICC* The conditional intraclass correlation coefficient is controlled for covariates

**Table 5:** Multilevel models of endotoxin and DM levels on EDC in households

Fixed effects	Category	Number (*n*)	Endotoxin				Domestic mite			
Variable			Median (IQR) [EU/m^2^/2wk]	Estimate	95% CI	*p* value	Median (IQR) [ng/m^2^/2 week**]**	Estimate	95% CI	*p* value
Intercept				529	(396–707)			533	(245–1162)	
Season	Spring	107	530 (246–1150)	1			374 (106–1174)	1		
	Summer	110	1521 (805–2406)	2.79	(2.26–3.45)	** < .0001**	704 (146–2929)	2.74	(1.88–4.01)	** < .0001**
	Autumn	98	642 (231–1300)	1.38	(1.11–1.72)	**0.0043**	583 (133–1423)	1.69	(1.14–2.50)	**0.0092**
	Winter	90	680 (358–1588)	1.65	(1.32–2.07)	** < .0001**	351 (164–876)	1.3		0.2
Persons in household	1–2	238	618 (300–1433)	1			275 (89–1187)	1		
	3	75	1115 (352–2406)	1.54	(1.08–2.20)	**0.016**	530 (109–1703)	1.73	(0.90–3.35)	0.1
	≥ 4	92	1220 (601–2331)	1.86	(1.31–2.62)	**0.0005**	682 (367–1541)	2.42	(1.25–4.68)	**0.0085**
Mite-allergic	No	284	922 (380–1860)	1						
resident	Yes	121	601 (334–1300)	0.65	(0.49–0.87)	**0.0035**				
Dog in	No	377	716 (349–1860)	1			476 (133–1541)	1		
household	Yes	28	1629 (666–4311)	1.85	(1.11–3.09)	**0.019**	190 (65–682)	0.3	(0.11–0.81)	**0.018**
Plants	No	189	934 (363–1811)	1						
	Yes	216	712 (354–1631)	0.8	(0.62–1.02)	0.069				
Days without room use	0	251	948 (387–1825)	1			441 (112–1337)	1		
	1–2.5	110	698 (401–1493)	0.91	(0.74–1.13)	0.39	587 (200–1916)	1.74	(1.18–2.56)	**0.0049**
	≥ 3	44	412 (193–1438)	0.7	(0.52–0.95)	**0.024**	218 (85–773)	0.82	(0.47–1.44)	0.49
Type of room	Sleeping	232					642 (183–2131)	1		
	Living	173					241 (102–740)	0.38	(0.20–0.69)	**0.0017**
Floor	Ground	150					480 (173–1406)	1		
	1. floor	126					635 (145–1957)	0.78	(0.38–1.60)	0.49
	≥ 2. floor	129					290 (54–980)	0.3	(0.15–0.59)	**0.0006**
Wet mopping	No	114					726 (233–1972)	1		
	Yes	291					369 (106–1217)	0.69	(0.46–1.03)	0.069

Random effects	Endotoxin	Domestic mite
Level-one variance (Sample)	0.56	1.81
Level-two variance (Room)	0.51	3.7
Conditional ICC	0.47	0.67

Influences with p values < 0.05 are printed in bold font. IQR: Interquartile range. Estimate: This is the transformed coefficient estimate exp (β) of the multilevel model. Conditional ICC: The conditional intraclass correlation coefficient is controlled for covariates

### Influence on endotoxin and allergen levels in homes

In the employees’ homes, endotoxin and DM concentrations also depended strongly on the season, but in contrast to the data in offices they were particularly high in summer (Table 5). In contrast, cat and dog allergen concentrations in households—without the respective pet—were significantly lower in summer than in winter (*p* = 0.012, *p* = 0.003, respectively, Table 6). Endotoxin and all allergen concentrations increased with the number of persons in the household. Furthermore, households with mite-allergic residents had significantly lower endotoxin concentrations, while no effect was observed on allergen concentrations. Households with dogs showed significantly higher endotoxin concentrations but lower DM levels, and bedrooms had significantly higher DM concentrations than living rooms. Rooms on the 2nd floor or above had significantly lower DM and Fel d 1 concentrations. The conditional ICC of the cat allergen Fel d 1 was only 17% compared to 77% for Can f 1, 67% for DM and 47% for endotoxin. Thus, a specific room was no strong determinant for the contamination with Fel d 1 in households without cats over the different seasons; whereas, the contamination of Can f 1 in households without dogs and the DM concentrations and endotoxin levels were influenced by the room.

**Table 6:** Multilevel models of cat and dog allergen levels on EDC in households without the respective pet

Fixed effects		Number (*n*)	Fel d 1				Number (*n*)	Can f 1			
Variable			Median (IQR) [ng/m^2^/2 week]	Estimate	95% CI	*p* value		Median (IQR) [ng/m^2^/2 wk]	Estimate	95% CI	*p* value
Intercept				1.66	(0.64–4.29)				0.09	(0.03–0.30)	
Season	Spring	86	4.15 (1.15–11.53)	1			98	1.46 (< 0.08–6.77)	1		
	Summer	87	3.16 (0.05–9.33)	0.57	(0.22–1.45)	0.23	103	< 0.08 (< 0.08–8.28)	0.23	(0.07–0.68)	**0.0084**
	Autumn	78	4.31 (1.38–9.23)	1.14	(0.44–2.99)	0.78	90	< 0.08 (< 0.08–9.23)	0.45	(0.15–1.41)	0.17
	Winter	76	4.42 (1.09–11.53)	1.93	(0.73–5.08)	0.18	86	1.46 (< 0.08–4.84)	1.24	(0.40–3.80)	0.71
Persons in household	1–2	206	3.65 (0.54–10.05)	1			222	< 0.08 (< 0.08–5.92)	1		
	3	54	5.27 (0.71–12.30)	1.45	(0.48–4.35)	0.51	70	< 0.08 (< 0.08–4.38)	0.88	(0.20–3.91)	0.86
	≥ 4	67	4.46 (1.72–10.77)	3.05	(1.10–8.48)	**0.033**	85	4.58 (< 0.08–10.77)	6.49	(1.56–27.0)	**0.01**
Plants	No						178	< 0.08 (< 0.08–5.92)	1		
	Yes						199	1.77 (< 0.08–9.23)	2.91	(0.98–8.66)	0.055
Floor	Ground	206	5.27 (1.69–14.61)	1							
	1. floor	54	3.69 (0.92–9.11)	0.5	(0.19–1.31)	0.16					
	≥ 2. floor	67	2.69 (0.28–6.69)	0.29	(0.11–0.80)	**0.016**					
Heating	Radiator						321	< 0.08 (< 0.08–5.02)	1		
	Underfloor						56	4.73 (< 0.08–14.83)	3.45	(0.67–17.7)	0.14

Random Effects	Fel d 1	Can f 1
Level-one variance (Sample)	9.23	11.19
Level-two variance (Room)	1.93	37.06
Conditional ICC	0.17	0.77

Influences with *p* values < 0.05 are printed in bold font. *IQR* Interquartile range. *Estimate* This is the transformed coefficient estimate exp (β) of the multilevel model. Conditional *ICC* The conditional intraclass correlation coefficient is controlled for covariates

## Discussion

### Comparing the concentration levels of allergens and endotoxins in employees' homes and offices

The initial question, whether the exposure at the office is higher than at home, can be answered for endotoxin and the mite allergens on the basis of the analysed EDC samples, that is, the concentrations were significantly lower at the office than at home. According to the models, households with three or more persons had endotoxin concentrations that were approximately three times higher than those at the office, while households with one or two persons were twofold higher. The difference between households and offices was even greater for mite allergens. The DM concentration was 26 times higher in living rooms and even 62 times higher in bedrooms than in offices. Thus, the mite allergen load in offices (median 27 ng/m^2^/2 week) differs significantly from the DM load that was previously quantified with the same method in day-care centres (median 364 ng/m^2^/week) (Sander et al. 2018). Interestingly, the DM concentrations measured in the EDC samples were even higher in the day-care centres than in household samples. The studies conducted in schools and homes in the Netherlands, which also recorded exposure by EDC samples and used the same quantification method for DM, found slightly lower DM concentrations (Krop et al. 2014), but higher endotoxin concentrations in schools than in households (Jacobs et al. 2013). However, due to the different methods used to determine endotoxin concentrations, the absolute values cannot be compared with our study.

In the case of cat and dog allergens, it is worthwhile to look at the results in a more differentiated manner. In households with these pets, the concentrations of cat or dog allergens were 3–4 orders of magnitude higher than in offices or households without these animals. However, the offices in which cat or dog owners worked differed significantly from the offices without pet owners. The concentration of the cat allergen, Fel d 1 was also significantly higher in offices where cat owners work than in households without cats, while the Can f 1 concentration in offices with dog owners was higher but not significantly different from households without dogs. So pet owners are exposed to much higher levels of allergens at home than in the office, while non-pet owners may experience higher exposure in the office than at home if they share their office with a pet owner. However, the median values in the offices were very low and also lower than, for example, the values in day-care centres or schools (Krop et al. 2014; Sander et al. 2018).

### Parameters that influence the allergen and endotoxin concentrations in offices

Regarding the concentration of cat or dog allergens in offices, a major influencing factor was the pet ownership of the employees sharing the room. On carpeted floors, the cat or dog allergen concentration was increased by a factor of more than three, and in EDC samples it was even increased by a factor of 9 and 17, respectively, when a cat or dog owner shared the room compared to offices without these pet owners. This is a strong indication that the animal allergens are transmitted to the office workplace by their owners. A possible transmission route is allergen-contaminated clothing or even hair, as has already been observed (Lucca et al. 2000; Krop et al. 2007; Karlsson and Renström 2005).

Another source for the transfer of allergens to the offices can be via contact with customers. In the floor samples, Can f 1 and Fel d 1 were two to three times higher in offices with customer contact compared to offices without customer contact. However, the influence from customers was significant only for Fel d 1, and was not found in the EDC samples from offices.

Other factors influenced the mite allergen concentration in offices. The DM concentration in EDC samples was elevated in rooms with ventilation or air conditioning, but not in the samples from the carpets in these rooms. It is possible that mite allergens are increasingly stirred up by air movement and settle on the passive collectors. However, no such effect was observed in cat and dog allergens that would have led to increased allergen concentrations in the EDC samples collected from ventilated or air-conditioned rooms. The major cat and dog allergens tend to be carried on small particles whereas dust mite allergens tend to be associated with large-size particles that settle rapidly (Grant et al. 2019). This could be a reason for the different influence of air conditioning on these allergen concentrations.

Endotoxins were lower in rooms with air conditioning compared to rooms with only window ventilation. One explanation would be endotoxin transfer from outside. This is also supported by data from Yoda et al. (2017), who observed a correlation between endotoxin in indoor and outdoor air, as well as increased indoor endotoxin concentrations when windows were open for more than one hour. The endotoxin concentration in offices was influenced significantly by the season; the concentration was significantly lowest in autumn. In contrast, the DM concentration in the offices was highest in autumn, as already observed in carpet and EDC samples in the day-care centre study (Sander et al. 2016b, 2018).

It is known that the reproduction of domestic mites is strongly dependent on sufficient humidity (Arlian et al. 2002), while the outdoor endotoxin concentration was inversely related to air humidity and increased with temperature (Carty et al. 2003). However, in our study relative air humidity in the office significantly increased the endotoxin levels in EDC samples when the season was considered as additional explanatory variable whereas humidity was no factor of influence on DM concentrations.

### Parameters influencing allergen and endotoxin concentrations in homes

In households, the endotoxin concentration increased with the number of users. This effect was also observed for all allergens in the households; in particular, households with four or more persons had higher endotoxin and allergen concentrations in the EDC samples than households with only one or two persons. Since the presence of dogs or cats in a household drastically increased the corresponding allergen levels, and other influencing factors hardly appeared to play a role in this case, only samples from households without the corresponding pets were considered in the models of animal allergens in households. Under this condition, a significantly lower concentration of Fel d 1, as well as DM was found in rooms on the second floor compared to the first floor. A significantly lower DM concentration was also observed in EDC samples collected from upper floors of schools in the Netherlands (Krop et al. 2014), but not in the homes of children and day-care workers in our previous study (Sander et al. 2018). Endotoxin was significantly elevated in households with dogs, as previously observed in U.S. homes (Thorne et al. 2009; Park et al. 2001). This was in contrast to the observation that DM concentrations were even significantly lower in households with dogs than those without. Households with mite-allergic residents had lower endotoxin concentrations, but no effect on DM concentrations was found. Neither measures for mite avoidance, nor floors without carpeting showed an effect on DM concentration in the EDC samples. This confirms previous findings using the same methodology (Sander et al. 2018). It also verifies that it is difficult to reduce airborne allergen concentration and the exposure to allergens (Punsmann et al. 2019), as was previously discussed in critical reviews on the limited success in the prevention of mite allergy (Tovey and Ferro 2012; Tovey and Marks 2011; van Boven et al. 2019). Custovic et al. (2019) stated that “the real question is not whether allergen avoidance is effective, but how to achieve a sufficient reduction in personal allergen exposure in real-life”.

## Conclusions

A limitation of our study is the observational nature of the study and the small number of companies where we could collect samples. No data on economical status of the companies and their employees was obtained. Since offices in one company had essentially uniform cleaning, ventilation and renovation parameters, an independent evaluation of these parameters was not possible. Therefore the generalization of this study may be limited.

In contrast, a major strength of our study is the high number of EDC samples and the parallel collection at homes and offices, during all four seasons. The comparison between the exposure at offices and private homes benefits from this. In our opinion, the comparatively simple and inexpensive method of allergen quantification using EDCs for the approximate assessment of airborne allergen exposure can make an important contribution to the improvement of intervention studies for allergy prevention.

In the case of legal case-by-case assessment of allergen exposure in the workplace, the personal collection of inhalable dust remains the method of choice for allergen quantification. In studies with many samples, sampling with personal pumps is usually too time-consuming and only covers a short period of time. Sampling of reservoir dust by vacuuming surfaces has been used in most studies so far. However, particles that are too large to be whirled up and inhaled are also recorded. Concentrations also depend on whether smooth floors, carpets, beds or furniture are vacuumed (Sander et al. 2016b, 2020). This makes a comparison between occupational and private exposures difficult due to different room furnishings. However, the allergen concentrations on the carpets in the offices as well as on the EDCs are quite low compared to day-care centres, schools or households (Engelhart et al. 2002; Krop et al. 2014; Sander et al. 2016b, 2018). The present study gives however an indication that employees without pets may be more highly exposed, particularly to cat allergens at their offices, than in their own household if they share an office with a pet owner. Both the EDC samples and the floor samples show significantly increased cat and dog allergen levels when respective pet owners share an office. In case of complaints at the office, employers and physicians should therefore consider a possible contamination by cat and dog allergens if a corresponding allergy and medical history exists.

## Supplementary Information

Below is the link to the electronic supplementary material.

Supplementary file1 (DOCX 22 KB) Explanatory variables and sample numbers in office models.

Supplementary file2 (DOCX 23 KB) Explanatory variables and sample numbers in household models.

Supplementary file3 (DOCX 28 KB) Multilevel models to Fig 1 and 2.

## Data Availability

The final data set is available here: https://ruhr-uni-bochum.sciebo.de/s/3qR1v8jxfTZ0JJQ.
