# Supplementary material for: Comparing the concentration levels of allergens and endotoxins in employees’ homes and offices
Source: Int Arch Occup Environ Health. 2021 Nov 5;95(3):573–88. doi: 10.1007/s00420-021-01794-9 (PMC8938351; doi:10.1007/s00420-021-01794-9)
Supplement: Supplementary file 1 — Supplementary file1 (DOCX 22 KB) Explanatory variables and sample numbers in office models. [file 420_2021_1794_MOESM1_ESM.docx]

S1 Table. Independent variables and sample numbers in office models

| ***Variable*** | ***Category*** | ***Statistical model: Type of independent variable*** | ***Vaccuumed floor samples (n)*** | ***EDC samples (n)*** |
| --- | --- | --- | --- | --- |
| Season | Spring | Within-rooms | 107 | 106 |
|  | Summer |  | 110 | 110 |
|  | Autumn |  | 110 | 110 |
|  | Winter |  | 110 | 110 |
| Number of employees in room | 1 | Between-rooms | 124 | 123 |
|  | 2-4 |  | 108 | 108 |
|  | 5-24 |  | 72 | 72 |
|  | 450 |  | 133 | 133 |
| Customer contact | Yes | Between-rooms | 256 | 256 |
|  | No |  | 181 | 180 |
| Air humidity | (continuous, starting at the minimum of air humidity) | Within-rooms | 437 | 436 |
| Employee with cat | Yes | Between-rooms | 261 | 260 |
|  | No |  | 176 | 176 |
| Employee with dog | Yes | Between-rooms | 221 | 221 |
|  | No |  | 216 | 215 |
| Height of room | ≤ 3 m | Between-rooms | 236 | 235 |
|  | > 3 m |  | 201 | 201 |
| Ventilation, renovation and cleaning | Windows to open, weekly cleaning, no floor renovation | Between-rooms | 116 | 116 |
|  | Windows to open, daily cleaning, no floor renovation |  | 28 | 27 |
|  | Windows to open, cleaning 1 - 2 days ago, floor renovation |  | 72 | 72 |
|  | Air conditioning, daily cleaning, no floor renovation |  | 20 | 20 |
|  | Air conditioning, daily cleaning, floor renovation |  | 137 | 137 |
|  | Ventilation system, cleaning 1 - 2 days ago, no floor renovation |  | 64 | 64 |
| Occupancy (percentage of room usage) † | (continuous) | Between-rooms |  | 436 |

†Usage during the 14 days of EDC sampling
