# Supplementary material for: Comparing the concentration levels of allergens and endotoxins in employees’ homes and offices
Source: Int Arch Occup Environ Health. 2021 Nov 5;95(3):573–88. doi: 10.1007/s00420-021-01794-9 (PMC8938351; doi:10.1007/s00420-021-01794-9)
Supplement: Supplementary file 2 — Supplementary file2 (DOCX 23 KB) Explanatory variables and sample numbers in household models. [file 420_2021_1794_MOESM2_ESM.docx]

S2 Table. Independent variables and sample numbers in household models

| ***Variable*** | ***Category*** | ***Statistical model: Type of independent variable*** | ***EDC samples (n)*** |
| --- | --- | --- | --- |
| Season | Spring | Within-rooms | 107 |
|  | Summer |  | 110 |
|  | Autumn |  | 98 |
|  | Winter |  | 90 |
| Persons in household | 1-2 | Within-rooms | 238 |
|  | 3 |  | 75 |
|  | ≥4 |  | 92 |
| Mite allergic resident | Yes | Within-rooms | 121 |
|  | No |  | 284 |
| Mite avoidance | Yes | Within-rooms | 219 |
|  | No |  | 186 |
| Frequent airing | Yes | Within-rooms | 180 |
|  | No |  | 225 |
| Cat in household | Yes | Within-rooms | 78 |
|  | No |  | 327 |
| Dog in household | Yes | Within-rooms | 28 |
|  | No |  | 377 |
| Plants | Yes | Within-rooms | 216 |
|  | No |  | 189 |
| Days without room use | 0 | Within-rooms | 251 |
|  | 1-2.5 |  | 110 |
|  | ≥ 3 |  | 44 |
| Type of room | Sleeping | Between-rooms | 232 |
|  | Living |  | 173 |
| Floor level | Ground | Between-rooms | 150 |
|  | 1^st^ floor |  | 126 |
|  | ≥ 2^nd^ floor |  | 129 |
| Heating | Radiator | Between-rooms | 344 |
|  | Underfloor |  | 61 |
| Cleaning up | Yes | Within-rooms | 371 |
|  | No |  | 34 |
| Vacuuming | Yes | Within-rooms | 382 |
|  | No |  | 23 |
| Wet mopping | Yes | Within-rooms | 291 |
|  | No |  | 114 |
| Sweeping | Yes | Within-rooms | 225 |
|  | No |  | 180 |
| Dusting | Yes | Within-rooms | 353 |
|  | No |  | 52 |
| Height of room | ≤ 2.4 m | Between-rooms | 101 |
|  | > 2.4 m |  | 304 |
| Floor coverage | no carpet | Within-rooms | 177 |
|  | ≤ 75% of floor with carpet |  | 129 |
|  | > 75% of floor with carpet |  | 99 |
| Upholstered furniture per 10 m² | (continuous) | Within-rooms | 405 |
