# Supplementary material for: Comparing the concentration levels of allergens and endotoxins in employees’ homes and offices
Source: Int Arch Occup Environ Health. 2021 Nov 5;95(3):573–88. doi: 10.1007/s00420-021-01794-9 (PMC8938351; doi:10.1007/s00420-021-01794-9)
Supplement: Supplementary file 3 — Supplementary file3 (DOCX 28 KB) Multilevel models to Fig 1 and 2. [file 420_2021_1794_MOESM3_ESM.docx]

S3 Tables. Multilevel models to Fig 1 and 2

Table A: Multilevel model of endotoxin on EDC in offices and households

| *Variable* | *Category* | *Number* | *Estimate* | *95% CI* | *p-value* |
| --- | --- | --- | --- | --- | --- |
| Intercept |  |  | 542 | (446 - 658) | <.0001 |
| Season | Spring | 213 | 1 |  | . |
|  | Summer | 220 | 1.698 | (1.45 - 1.99) | **<0.0001** |
|  | Autumn | 208 | 0.995 | (0.85 - 1.17) | 0.9478 |
|  | Winter | 200 | 1.165 | (0.99 - 1.37) | 0.0663 |
| Type of room and number of persons | Office: 1-4 persons | 231 | 0.571 | (0.45 - 0.73) | **<0.0001** |
|  | Office: >=5 persons | 205 | 0.642 | (0.41 - 0.997) | **0.0485** |
|  | Household: 1-2 persons | 238 | 1 |  | . |
|  | Household: >=3 persons | 167 | 1.673 | (1.3 - 2.16) | **<0.0001** |

Influences with p-values < 0.05 are printed in bold font. Estimate: This is the transformed coefficient estimate exp (ß) of the multilevel model

Table B: Multilevel model of DM on EDC in offices and households

| *Variable* | *Category* | *Number (< range)* | *Estimate* | *95% CI* | *p-value* |
| --- | --- | --- | --- | --- | --- |
| Intercept |  |  | 245 | (141 - 428) | **<0.0001** |
| Season | Spring | 213 (20) | 1 |  |  |
|  | Summer | 220 (63) | 0.50 | (0.33 - 0.76) | **0.0013** |
|  | Autumn | 208 (24) | 1.46 | (0.96 - 2.23) | 0.0783 |
|  | Winter | 200 (32) | 0.77 | (0.50 - 1.18) | 0.2226 |
| Type of room | Living room | 173 (6) | 1 |  |  |
|  | Sleeping room | 232 (9) | 2.39 | (1.25 - 4.58) | **0.0085** |
|  | Office | 436 (127) | 0.04 | (0.02 - 0.07) | **<0.0001** |

Influences with p-values < 0.05 are printed in bold font. Estimate: This is the transformed coefficient estimate exp (ß) of the multilevel model

Table C: Multilevel model of Fel d 1 in EDC samples in offices and households

| *Variable* | *Category* | *Number (< range)* | *Estimate* | *95% CI* | *p-value* |
| --- | --- | --- | --- | --- | --- |
| Intercept |  |  | **1.33** | (0.81 - 2.17) | 0.2554 |
| Season | Spring | 213 (24) | **1** |  |  |
|  | Summer | 220 (44) | **0.42** | (0.26 - 0.67) | **0.0003** |
|  | Autumn | 208 (21) | **1.66** | (1.03 - 2.68) | **0.0359** |
|  | Winter | 200 (14) | **1.60** | (0.99 - 2.59) | 0.0542 |
| Cat ownership | Household without cat | 327 (55) | **1** |  |  |
|  | Household with cat | 78 (0) | **962** | (388 - 2385) | **<0.0001** |
|  | Office without cat owner | 176 (37) | **0.46** | (0.22 - 0.94) | **0.0338** |
|  | Office with cat owner | 260 (11) | **4.89** | (2.02 - 11.84) | **0.0004** |

Influences with p-values < 0.05 are printed in bold font. Estimate: This is the transformed coefficient estimate exp (ß) of the multilevel model

Table D: Multilevel model of Can f 1 in EDC samples in offices and households

| *Variable* | *Category* | *Number (<range)* | *Estimate* | *95% CI* | *p-value* |
| --- | --- | --- | --- | --- | --- |
| Intercept |  |  | 0.31 | 0.15 - 0.63 | **0.0012** |
| Season | Spring | 213 (87) | 1 |  |  |
|  | Summer | 220 (137) | 0.14 | 0.07 - 0.29 | **<0.0001** |
|  | Autumn | 208 (108) | 0.41 | 0.20 - 0.82 | **0.0123** |
|  | Winter | 200 (68) | 2.03 | 1.02 - 4.06 | **0.0441** |
| Dog ownership | Household without dog | 377 (190) | 1 |  |  |
|  | Household with dog | 28 (0) | 5946 | 860 - 41.1 | **<0.0001** |
|  | Office without dog owner | 215 (141) | 0.16 | 0.06 - 0.45 | **0.0006** |
|  | Office with dog owner | 221 (69) | 3.21 | 0.64 - 16.1 | 0.1564 |

Influences with p-values < 0.05 are printed in bold font. Estimate: This is the transformed coefficient estimate exp (ß) of the multilevel model
